# 576 Comparison of Survival Benefit Between Direct Emergency Department Admissions and Inter-hospital Transfers in Burn Victims

**DOI:** 10.1093/jbcr/irac012.204

**Published:** 2022-03-23

**Authors:** Ian F Hulsebos, Zachary J Collier, Haig A Yenikomshian, Justin Gillenwater

**Affiliations:** Keck School of Medicine of USC, Pasadena, California; Keck School of Medicine, University of Southern California, Los Angeles, California; USC/LAC+USC Medical Center, Los Angeles, California; USC/LAC+USC Medical Center, Los Angeles, California

## Abstract

**Introduction:**

Severe burn injuries create systemic insults that are life threatening and require immediate stabilization, resuscitation, and, potentially, emergent surgery. Non-burn emergencies, such as stroke, have shown improved survival benefit and outcomes when patients are immediately transferred to specialized facilities. Our objective was to compare the outcomes between burn patients who were transferred to the burn unit from outside hospitals (HT) to controls who were directly admitted from our own emergency department (ED). We hypothesized that HT patients were at increased risk for mortality, wound healing complications, and infectious sequelae relative to the ED cohort.

**Methods:**

A matched retrospective cohort study from July 1, 2015 to November 1, 2019 was performed at an ABA-verified burn center. HT patients were identified and matched with ED patients by age and percent total body surface area burned (TBSA). HT and ED cohorts were compared as a whole and then stratified into groups according to % TBSA burned (< 10%, 11-20%, 21-40%, and > 40%). Patient and burn characteristics were recorded. The primary outcome was mortality, and secondary outcomes included total length of stay (LOS), ICU requirement and complications.

**Results:**

A total of 410 HT and 377 ED patients were identified. There were no significant differences in age (P=0.17), burn severity (TBSA: P=0.27; full thickness burns: P=0.13), and inhalation injury (P=0.29). There were no demographic differences when comparing cohorts according to TBSA. For the primary outcome, there were no significant differences in mortality in the cohorts at large (P=0.48) nor between the groups when stratifying for TBSA (< 10% : P=0.35; 11-20% : P=0.44; 21-40% : P=0.30; >40% : P=0.26). For the secondary outcomes at large, there was no significant difference in LOS (P=0.35), ICU requirement (P=0.17), or wound and infectious complications (P=0.14). HT patients at large, however, spent less time in the ICU (P=0.03).

**Conclusions:**

There was no significant difference in overall mortality rates between the HT and ED groups regardless of TBSA. Additionally, there was no difference in hospital course between the cohorts except for longer ICU stays in those admitted directly from the ED. Once the transfer process is initiated, our unit maintains close physician-to-physician communication with the transferring facility throughout the transfer process, including guiding initial resuscitation efforts. This may play a role in the parity of outcomes between groups.

